# Myotubes Formed De Novo by Myoblasts Injected into the Scar of Myocardial Infarction Persisted for 16 Years in a Patient: Importance for Regenerative Medicine in Degenerative Myopathies

**DOI:** 10.1002/sctm.18-0202

**Published:** 2018-12-01

**Authors:** Daniel Skuk, Jacques P. Tremblay

**Affiliations:** ^1^ Axe Neurosciences Research Center of the CHU de Quebec—CHUL Quebec Canada

A recent clinical report of Dr. Menasché’s group 1 showed that the heart of a patient, who received injections of autologous myoblasts in the infarction scar 16 years before, contained accumulations of myotubes derived from the graft. This observation was possible because the patient underwent heart transplantation for end‐stage heart failure, thus the cell‐injected heart was analyzed after resection. In a commentary published simultaneously 2, Dr. Gnecchi evaluated the results focused on cell therapy of cardiac pathologies, concluding that this report should close the saga of clinical trials of intracardiac myoblast transplantation started in 2001, when Menasché’s group reported the first patient receiving this experimental approach to treat ischemic heart failure 3.

We believe that is also necessary to highlight the importance of this finding for a potential treatment of skeletal muscle degenerative disorders, among which Duchenne muscular dystrophy (DMD) is the emblematic case. In DMD, the genetic deficiency of dystrophin, a subsarcolemmal protein, causes relentless loss of myofibers and replacement by fibrosis and adipocytes 4. In terminal phases, of the original muscles remain only vestiges composed of disorganized connective and adipose tissue with few, if any, atrophic myofibers.

Much experimental progress was made in gene therapy to restore the expression of dystrophin in DMD 5, an objective needed to stop or slow down the disease progression. However, very little has been done to improve DMD patients with advanced disease, in which we need to restore not only dystrophin but the muscle tissue that was irreversibly lost (Fig. 1). To create new myofibers, cell therapy in a regenerative medicine context seems the only option.

**Figure 1:**
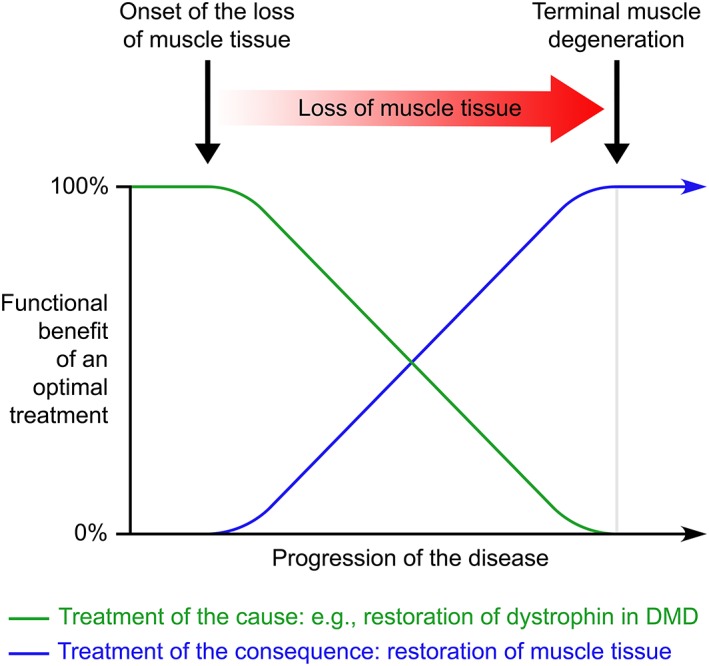
The graph shows schematically the changes of treatment priorities in degenerative muscular diseases as Duchenne muscular dystrophy (DMD). Although the treatment of the cause (e.g., restoring dystrophin in DMD myofibers) is the most relevant objective when the muscle tissue is more or less preserved, the irreversible deterioration of the muscles over time implies that other strategy becomes progressively more necessary to help the patient: to regenerate the lost tissue. The same scheme would apply, in fact, to any degenerative disease in which there is irreversible destruction of tissue.

Several years ago, we reported histological observations suggesting that normal myoblasts transplanted in DMD patients formed abundant new small dystrophin‐positive myofibers in the fibrotic tissue of some muscles 6. The histology coincided with the neoformation of myofibers after myoblast transplantation in mouse muscles 7 and with the process of intramuscular myoblast engraftment described in macaques 8, but we found no method to irrefutably confirm that these small dystrophin‐positive myofibers were formed de novo by the grafted myoblasts, and were not due to fusion of the grafted cells with pre‐existing atrophic myofibers.

First confirmation that this neoformation was possible in humans came in fact from Dr. Menasche's group, which previously reported myotubes in a myocardial infarction scar 17 months after autologous myoblast transplantation 9. Since myotubes do not appear spontaneously in the heart, there is no doubt that they were formed de novo by the grafted myoblasts. Now, the new report provides not only further clinical confirmation that transplanted myoblasts fused together to form myotubes in fibrotic tissue but, importantly, that these myotubes survived outside their normal tissular niche for as long as 16 years (what could be extrapolated as indefinitely as long as there is no immune rejection 10, which occurs under allogeneic conditions with a sustained immunosuppression or following autologous transplantation).

Certainly, improving this result, that is, moving from producing a group of myotubes in a fibrotic tissue to producing a contractile functional muscle, implies several challenges. Myotubes must be sufficiently long, receive adequate innervation, develop into mature myofibers, and fibrosis must be reversed. Achieving those goals requires the investigation of many aspects. Interestingly, the images in the article 1 suggest that the neoformed myotubes are aligned with each other, which is essential for a coordinate contraction.

Therefore, we consider that the report of Dr. Menasché’s group 1 is stimulant for the field of regenerative medicine in the skeletal muscle, which seems the only hope to improve patients with advanced muscle degeneration.

## Disclosure of Potential Conflicts of Interest

The authors indicated no potential conflicts of interest.
